# Bridging neuroscience and AI: a survey on large language models for neurological signal interpretation

**DOI:** 10.3389/fninf.2025.1561401

**Published:** 2025-06-18

**Authors:** Sreejith Chandrasekharan, Jisu Elsa Jacob

**Affiliations:** ^1^Freelance Researcher, Trivandrum, Kerala, India; ^2^Department of Electronics and Communication Engineering, Sree Chitra Thirunal College of Engineering, Trivandrum, Kerala, India

**Keywords:** electroencephalogram, large language model, LLM, BERT, GPT

## Abstract

Electroencephalogram (EEG) signal analysis is important for the diagnosis of various neurological conditions. Traditional deep neural networks, such as convolutional networks, sequence-to-sequence networks, and hybrids of such neural networks were proven to be effective for a wide range of neurological disease classifications. However, these are limited by the requirement of a large dataset, extensive training, and hyperparameter tuning, which require expert-level machine learning knowledge. This survey paper aims to explore the ability of Large Language Models (LLMs) to transform existing systems of EEG-based disease diagnostics. LLMs have a vast background knowledge in neuroscience, disease diagnostics, and EEG signal processing techniques. Thus, these models are capable of achieving expert-level performance with minimal training data, nominal fine-tuning, and less computational overhead, leading to a shorter time to find effective solutions for diagnostics. Further, in comparison with traditional methods, LLM's capability to generate intermediate results and meaningful reasoning makes it more reliable and transparent. This paper delves into several use cases of LLM in EEG signal analysis and attempts to provide a comprehensive understanding of techniques in the domain that can be applied to different disease diagnostics. The study also strives to highlight challenges in the deployment of LLM models, ethical considerations, and bottlenecks in optimizing models due to requirements of specialized methods such as Low-Rank Adapation. In general, this survey aims to stimulate research in the area of EEG disease diagnostics by effectively using LLMs and associated techniques in machine learning pipelines.

## 1 Introduction

Artificial intelligence and machine learning techniques have greatly contributed to the field of EEG signal processing. The emergence of Large Language Models (LLMs) to interpret and understand complex brain activity patterns is a new era in EEG signal processing. Electroencephalography (EEG) or brain signal is one of the best techniques for measuring neural activity and provides a vast amount of temporal data that requires efficient algorithms for analysis and thereby extracting meaningful insights and information. As EEG signals are non-invasive, more portable, have greater potential for use, and apply to a wider population.

EEG analysis can be performed using different methods:

(i) **Representation learning in EEG analysis:** It is the basic step in EEG analysis that can extract relevant features from EEG signals that are useful for identifying certain states or pathologies. It is performed using self-supervised learning methods to develop biomarkers for various pathologies. This analysis technique can be applied to huge brain signal data.
(ii) **Discriminative EEG analysis:** As the name suggests, this analysis technique is employed for discrimination and for distinguishing between various groups like disease and normal, based on various patterns in EEG signals. This can be done using advanced architectures such as Foundation Models, LLMs, and Graph Neural Networks (GNNs). This architecture efficiently captures the EEG patterns, which are capable of discrimination and are crucial for learning complex neural processes.
(iii) **Generative EEG analysis:** Generative EEG analysis refers to a set of techniques that aims to understand and model the underlying processes that cause electrical activity in the brain and generate EEG signals. Unlike traditional EEG analysis, which focuses on identifying patterns or abnormalities in the recorded EEG data, generative approaches seek to explain how these signals are produced by the brain and how they relate to cognitive or neural processes. Generative methods aim to generate new modalities or signal data from EEG signals. Innovative approaches such as diffusion produce images or text from EEG data, providing novel approaches to the understanding and visualization of brain activity.

The application of LLMs to EEG interpretation addresses several critical challenges in neuroscience research and clinical practice. First, EEG data is inherently complex, containing various frequency bands, spatial relationships, and temporal patterns that cannot be completely explored by traditional analysis methods. LLMs, with their ability to process sequential data and identify long-range dependencies, offer a promising approach to understanding these intricate patterns within the EEG signal. Secondly, the medical interpretation of EEG often relies heavily on expert knowledge and pattern recognition skills developed over many years. The capacity of LLMs to learn from large datasets of annotated EEG recordings and medical reports could help bridge this expertise gap and support clinical decision making.

Furthermore, LLMs are good at understanding context and generating natural language descriptions, which makes them particularly valuable for translating complex EEG patterns into clinically relevant insights. This LLM capability can revolutionize the way EEG signals are analyzed and neurological findings are made and communicated between healthcare providers and researchers. This can potentially improve diagnostic accuracy and treatment planning. The ability of such models to process multimodal inputs also gives new possibilities for integrating EEG data with other clinical information, thereby creating a more comprehensive understanding of various neurological pathologies.

Generative Large Language Models (LLMs) often present several benefits compared to pre-trained Transformer language models (Kalajdzievski, 2024). Firstly, many generative LLMs can perform tasks without requiring explicit fine-tuning on annotated datasets, leading to considerable savings in time and resources associated with data annotation. Secondly, these models frequently overcome the limitation of a fixed maximum input length, enabling the processing of longer sequences of text. Thirdly, task-specific behavior in generative LLMs is often achieved through prompt engineering, which can be a more efficient approach than extensive hyperparameter optimization typically needed for pre-trained Transformer models.

LLMs are less susceptible to data imbalance issues due to the vast pre-training they receive, covering domain knowledge, signal analysis, and related methodologies. Fine-tuning methods in LLMs, such as PEFT, freeze base model weights, and preserve core knowledge. This approach prevents catastrophic overwriting compared to training approaches used with traditional deep learning methods. Hence, LLMs are less dependent on perfectly balanced training datasets compared to traditional models.

Recent advances in LLM architectures and training techniques have made these applications more feasible. The development of specialized attention mechanisms and temporal embedding methods has enhanced their ability to process time-series data like EEG. Additionally, the success of transfer learning in various domains suggests that pre-trained language models could be effectively utilized for neurological signal interpretation, thereby reducing the amount of labeled data required for specific applications. However, applying LLMs to EEG analysis also presents unique challenges that must be addressed. It includes the need for appropriate data representations, integration of domain-specific knowledge, and development of interpretable models that can provide clinically meaningful outputs. Understanding these challenges and potential solutions is crucial to advance the field and realize the full potential of LLM in neuroscience. This survey makes an effort to consolidate the major studies related to applying LLM in the context of EEG signal interpretation.

### 1.1 Understanding neural signaling

Classical research in the neurological sector is concentrated on advancements for diagnosing particular conditions, and the majority of this research is centered on handling neurological signals individually. There is a clear need to bridge the gap and pave the way toward more generalized neurological signal processing paradigms that can be applied to the broader context of neuroscience. Although early applications of LLMs focused on individual neural signals, mainly EEG and fMRI, the focus is now increasingly shifting toward unified frameworks capable of handling a wider range of neurological signals beyond these popular modalities. The following subsections give an overview of neurological signals that are considered in this study and inter-relations between them (Gentile and Barragan, 2023; Hong et al., 2018; Baghdadi et al., 2025; Chaudhary, 2025).

#### 1.1.1 EEG signals

Electroencephalography (EEG) signals are used to record the electrical activity of the brain via electrodes placed on the scalp. This non-invasive method captures the summation of postsynaptic potentials of numerous of neurons firing synchronously in the cerebral cortex. The resulting EEG signal reflects the ongoing electrical activity and exhibits its changes in response to various stimuli or pathological states. Small metal or silver/silver chloride electrodes are attached to the scalp for EEG recording using standard placements like the international 10–20 system. These electrodes detect minute voltage fluctuations on the scalp, which are then amplified and digitized by the EEG machine. The raw EEG data is typically formatted as a time series of voltage values for each electrode, representing the electrical potential difference between the recording electrode and a reference electrode over time. A significant strength of EEG is its high temporal resolution, as it can capture rapid changes in brain activity on the order of milliseconds, making it excellent for studying the timing of neural events. EEG also provides a direct measure of neuronal activity, offering a real-time window into brain function. Compared to other neuroimaging techniques like MEG and fMRI, EEG equipment and operational costs are generally lower, making it more accessible for research and clinical applications (Baghdadi et al., 2025). Furthermore, the non-invasive nature of EEG, with electrodes placed on the scalp surface, poses no surgical or internal risks to the subject. EEG systems can also be relatively compact and portable, allowing for recordings in various settings. However, EEG suffers from low spatial resolution because the electrical signals recorded on the scalp are blurred and attenuated as they pass through the skull and scalp, making it difficult to precisely localize the sources of neural activity within the brain. EEG signals are also highly susceptible to various artifacts originating from physiological sources (e.g., eye blinks, muscle movements, and heart activity) and external sources (e.g., electrical noise), requiring careful preprocessing. Finally, EEG is primarily sensitive to activity in the superficial layers of the cortex and has difficulty detecting activity originating from deeper brain structures.

#### 1.1.2 MEG signals

Magnetoencephalography (MEG) is a non-invasive neuroimaging technique that measures the magnetic fields produced by electrical activity in the brain. These magnetic fields, generated by the flow of ionic currents within neurons, are extremely weak and are detected by highly sensitive superconducting quantum interference devices (SQUIDs) housed in a cryogenic dewar that does not touch the patient's head. MEG is particularly sensitive to neuronal currents that are tangential to the scalp, making it complementary to EEG which is more sensitive to radial currents. During a MEG recording, the subject sits or lies down in a magnetically shielded room to minimize external magnetic interference. The dewar containing the SQUID sensors is positioned around the head, capturing the minute magnetic field changes. Simultaneously, the subject's head position relative to the sensors is often tracked using head position indicator (HPI) coils. The raw MEG data consists of a time series of magnetic field measurements for each sensor. Similar to EEG, MEG data is susceptible to artifacts from various sources, including environmental magnetic noise, movement of the subject, and physiological signals like heartbeats and eye blinks (Cuffin and Cohen, 1979). Magnetoencephalography (MEG) measures the magnetic fields produced by the electrical activity of the brain. These magnetic fields are less distorted by the skull and scalp compared to the electrical potentials measured by EEG. Similar to EEG, MEG offers excellent temporal resolution, capable of tracking rapid neural events in the millisecond range. MEG also directly measures the electromagnetic consequences of neuronal activity, providing a real-time assessment of brain function. A key advantage of MEG over EEG is its better spatial resolution because magnetic fields are less distorted by intervening tissues, allowing for more accurate localization of neural sources. However, MEG systems are significantly more expensive to purchase, maintain (due to the need for cryogenic cooling of the sensors), and operate compared to EEG. The use of MEG is also limited to specialized facilities due to the requirement of magnetically shielded rooms to minimize interference from external magnetic fields (Baghdadi et al., 2025). Despite shielding, MEG recordings can still be affected by subtle magnetic noise from the environment or even movement of metallic objects near the scanner.

#### 1.1.3 fMRI signals

Functional Magnetic Resonance Imaging (fMRI) is a neuroimaging technique that measures brain activity by detecting changes in blood flow and oxygenation. The underlying principle is neurovascular coupling, which posits that local neural activity is accompanied by changes in regional cerebral blood flow (rCBF) and blood oxygenation. fMRI most commonly utilizes the blood-oxygen-level-dependent (BOLD) contrast, which is sensitive to the ratio of oxygenated to deoxygenated hemoglobin in the blood. During an fMRI scan, the subject lies inside a strong magnetic field. Radio frequency pulses are applied, causing protons in the brain tissue to align and then relax, emitting signals that are detected by the MRI scanner. For fMRI, specific pulse sequences are used to make the images sensitive to the BOLD signal. A series of 3D brain volumes are acquired over time, capturing the dynamic changes in blood oxygenation related to neural activity. The raw fMRI data is a 4D dataset (3 spatial dimensions + time), where each voxel (volumetric pixel) contains a time series of signal intensity values. Functional Magnetic Resonance Imaging (fMRI) is a neuroimaging technique that measures brain activity by detecting changes in blood flow and oxygenation using a strong magnetic field. The most common method, Blood-Oxygen-Level-Dependent (BOLD) fMRI, relies on the different magnetic properties of oxygenated and deoxygenated hemoglobin. fMRI offers the highest spatial resolution among these four techniques, allowing for detailed mapping of brain activity down to the millimeter level. It also provides complete brain coverage in a single scan, offering a complete view of neural activity in different regions, and unlike EEG and fNIRS, fMRI is sensitive to activity in both the cortical and subcortical structures (deep brain) (Baghdadi et al., 2025). However, the hemodynamic response measured by fMRI is relatively slow, peaking several seconds after the onset of neural activity, which limits its ability to precisely track the timing of rapid neural events, resulting in low temporal resolution. fMRI scanners are very expensive to purchase, install, and operate, requiring specialized infrastructure and trained personnel, and they are non-portable, being large, stationary pieces of equipment. A significant limitation of fMRI is the requirement for participants to remain very still during scans to avoid motion artifacts, which can significantly degrade the image quality, posing challenges for certain populations.

#### 1.1.4 fNIRS signals

Functional Near-Infrared Spectroscopy (fNIRS) is a non-invasive neuroimaging technique that measures brain activity by assessing changes in the concentration of oxygenated hemoglobin (HbO) and deoxygenated hemoglobin (HbR) in the cerebral cortex. fNIRS utilizes the principle of neurovascular coupling, similar to fMRI, but uses near-infrared light to penetrate the scalp and skull. Changes in neural activity may lead to changes in blood flow and oxygen consumption, which in turn alter the absorption and scattering of the near-infrared light that passes through the brain tissue. An fNIRS system typically consists of light sources that emit near-infrared light at one or more wavelengths (typically between 700 and 900 nm) and detectors (photodiodes) placed on the scalp. The sources and detectors are arranged in optodes, which are positioned on the scalp using a cap or a custom-made holder. The light emitted by the sources travels through the head tissue and is partially absorbed and scattered before reaching the detectors. The intensity of the detected light at each wavelength is measured over time. The raw fNIRS data consists of time series of light intensity measurements for each source-detector pair (channel) and each wavelength. This raw data is then converted to changes in optical density. Functional Near-Infrared Spectroscopy (fNIRS) is an optical neuroimaging technique that measures brain activity by assessing changes in the concentration of oxygenated hemoglobin (HbO) and deoxygenated hemoglobin (HbR) in the cerebral cortex. It utilizes the principle that neural activity is coupled with changes in local blood flow. fNIRS offers the advantage of being portable, with devices that are generally lightweight and allowing for measurements in more naturalistic settings and with participants who may not be able to tolerate other imaging modalities (Hong et al., 2018). Due to its portability and tolerance to some movement, fNIRS is suitable for studying brain activity during tasks involving movement. The non-invasive nature of fNIRS, using light shone onto the scalp and detected by sensors, is another benefit, and compared to MEG and fMRI, fNIRS systems are often considered easier to set up and operate. However, fNIRS has relatively low spatial resolution compared to fMRI and even MEG, as the scattering of light in the tissue limits the precision of source localization (Gentile and Barragan, 2023). The depth penetration of near-infrared light is also limited, primarily allowing measurement of activity in the superficial layers of the cortex. As fNIRS measures brain activity indirectly through hemodynamic changes, which are slower than the direct neuronal activity measured by EEG and MEG, its temporal resolution is also limited compared to electrophysiological methods. Finally, the presence of hair and variations in scalp and skull thickness can affect the light transmission and signal quality, requiring careful consideration during setup and analysis, and a baseline scalp condition is typically needed for reliable measurements.

### 1.2 Overview of large language models

Advanced language models with huge parameter sizes and remarkable learning capacities are known as large language models, or LLMs. The self-attention module in Transformer (Vaswani et al., 2017) is the fundamental component of many LLMs, including GPT-3 (Floridi and Chiriatti, 2020) and GPT-4.

A crucial component of LLMs is in-context learning (Brown et al., 2020), in which the model is trained to produce text based on a specified context or prompt. As a result, LLMs can produce responses that are more logical and pertinent to the situation, which makes them appropriate for conversational and interactive applications. Another essential component of LLMs is Reinforcement Learning from Human Feedback (RLHF) (Christiano et al., 2017). By using human-generated replies as rewards, this technique fine-tunes the model, enabling it to learn from its errors and gradually enhance its performance. Prompt engineering is a popular method of communicating with LLMs in which users build and provide certain prompt messages to direct LLMs to produce the required responses or perform particular tasks (White et al., 2023; Clavié et al., 2023; Zhou et al., 2022). People can participate in dialogue interactions, which involve speaking with LLMs in natural language, or question-and-answer interactions, in which they ask the model questions and get replies. In summary, LLMs have transformed NLP and have the potential for several uses thanks to their Transformer architecture, in-context learning, and RLHF capabilities.

#### 1.2.1 Bidirectional encoder representations from transformers

Bidirectional Encoder Representations from Transformers (BERT) introduced a deep, bidirectional, unsupervised language representation. BERT considers the entire context of a word, both preceding and succeeding, during training, unlike previous models, which process text sequentially (Koroteev, 2021). This enables the model to capture rich semantic and syntactic information, leading to significant performance improvements in various NLP tasks. This powerful understanding is further enhanced by BERT's pre-training process, which utilizes two unsupervised learning objectives. Firstly, Masked Language Modeling (MLM) forces the model to deeply understand language semantics by randomly masking words in the input and training it to predict the masked words based on the surrounding context. Secondly, Next Sentence Prediction (NSP) improves the model's ability to understand discourse by training it to predict whether two given sentences are consecutive in the original text, thereby capturing crucial sentence-level relationships.

BERT's architecture is based on the Transformer model, which utilizes self-attention mechanisms to capture complex relationships between words. It consists of multiple layers of stacked transformer blocks, each containing a Multi-Head Self-Attention and Position-wise Feed-Forward Network (FFN) (Devlin et al., 2018; Hao et al., 2019). Multi-head self-attention allows the model to attend to different parts of the input sequence simultaneously, capturing diverse relationships between words. FFN introduces non-linearity and allows the model to learn complex representations. Two unsupervised pre-training tasks are used by BERT: Next Sentence Prediction, which asks the network to determine whether two sentences are consecutive, and Masked LM, in which some words are masked and the network infers their meaning from context.

The main limitations of BERT include high computational cost, requiring significant resources for pre-training, and difficulty in handling very long sequences due to its fixed maximum sequence length. It is challenging to fine-tune the BERT model for some specific tasks, which may require careful tuning of hyperparameters.

#### 1.2.2 GPT-1

There has been a long history behind GPT-1 dating back to the groundbreaking paper “Attention is all you need” (Vaswani et al., 2017). According to it, the Transformer is divided into two parts: encoder and decoder, both of which perform Multi-Head Self Attention, though the encoder is able to observe information from the entire source sequence while the decoder does not. Similar to filling in the gaps, the Bert model adjusts the encoder and uses context to forecast the missing intermediate phrases when creating pre-training tasks. GPT-1 also executes masked multi-head self-attention by using a decoder, which anticipates the subsequent context based on the preceding context. Making context predictions from a huge corpus of data is the pre-training phase. The final token's embedding is fed into the prediction layer, which fits the downstream data's label distribution after the model has been trained using downstream data during the fine-tuning stage. The model's accuracy and generalization abilities improve as the number of layers increases. Zero-shot learning is a built-in feature of GPT-1 and as the model gets bigger, so does this capability, which leads to the development of later GPT models.

#### 1.2.3 GPT-2

Based on the Transformer architecture for language modeling, GPT-2 is an improved version of GPT-1. Large amounts of unlabeled data can be used to train models with GPT-2, and fine-tuning improves model performance and optimizes it for downstream tasks. GPT-2 places more focus on the language model in a zero-shot scenario, when the model hasn't been trained or optimized for downstream tasks before being used. GPT-1 often relies on fine-tuning, and adjusting the model's parameters specifically for each downstream task. This typically involves introducing special tokens, such as start and separator symbols, to guide the model's understanding of the task at hand. In contrast, GPT-2 emphasizes zero-shot learning, aiming to perform tasks without explicit fine-tuning. This necessitates a different approach to task specification. Instead of modifying the model, GPT-2 primarily modifies the input sequences.

GPT-2 significantly scales up the Transformer architecture, boasting 48 layers and 1.5 billion parameters, compared to GPT-1's 12 layers and BERT's 24. This scaling necessitates a massive training dataset, derived from WebText after basic data cleaning. Research suggests that larger models require more data to reach their full potential, and current models, including GPT-2, are likely still under-trained (Radford et al., 2019). Unlike BERT, which employs bidirectional transformers, GPT-2 utilizes unidirectional transformers, mirroring the sequential nature of language generation. Furthermore, GPT-2 adopts a novel multi-tasking approach during pre-training. Instead of focusing on a single objective, it learns across multiple tasks simultaneously, ensuring that the model converges effectively. Notably, the core Transformer parameters are shared across these tasks, promoting efficient learning and enhancing generalization. This multi-tasking strategy, inspired by MT-DNN (Liu et al., 2020), empowers GPT-2 to achieve impressive performance even without task-specific fine-tuning.

#### 1.2.4 GPT-3

GPT-3 primarily focuses on the idea of a universal language model excluding traditional fine-tuning. To address the computational challenges associated with its massive 175 billion parameters, GPT-3 incorporates the sparse attention mechanism from Sparse Transformers (Floridi and Chiriatti, 2020). This technique reduces computational load by selectively attending to relevant parts of the input sequence. For downstream tasks, GPT-3 employs a few-shot learning approach, demonstrating remarkable performance with just a few examples. This highlights the significant impact of model size on few-shot learning capabilities. The GPT-3 architecture is identical to the GPT 2, except the transformer layers have dense and sparse attention (Child et al., 2019; Radford et al., 2019). GPT-3 employs the gradient noise scale as in (McCandlish et al., 2018) to determine the batch size during training, demonstrating that big models may train on larger batch sizes with a lower learning rate. In general, GPT-3 raises model parameters to 175B, demonstrating that large language models improve with the scale and are competitive with the fine-tuned models.

One important feature of GPT-3 is its capacity for in-context learning. By merely supplying examples within the input sequence, in-context learning provides few-shot performance, in contrast to traditional fine-tuning, which updates model parameters based on downstream task examples. As the number of instances increases, this “prompting” strategy shows a notable performance improvement. But after eight shots, the effect of more examples decreases and, after ten rounds, is insignificant.

#### 1.2.5 GPT-4

In comparison to GPT-3, GPT-4 has more than a trillion parameters and greatly enhances the GPT model scale and training methods. The GPT-4 model may produce text more accurately and naturally by employing a novel training method called Reinforcement Learning from Human Feedback (RLHF). To train through reinforcement learning, RLHF combines pre-training and fine-tuning techniques, having conversations with human operators. This increases GPT-4's performance on particular tasks and strengthens its understanding of context and questions (Nori et al., 2023; Wang et al., 2023). GPT-4 generally employs the same pre-training, prompting, and prediction-based training methodology as ChatGPT. Three noteworthy improvements are introduced in GPT-4: (1) Using a rule-based reward model (RBRM); (2) Including multi-modal prompt learning to accommodate different prompts; (3) Including a chain of thought mechanism to improve overall coherence in thinking. GPT-4 is a strong multimodal model that can interpret text and image input, producing text outputs that rank among the top 10% of test takers. On conventional benchmarks, the GPT-4 language model performs better than most cutting-edge NLP systems (Liu et al., 2021; Chang et al., 2023).

#### 1.2.6 Claude

Anthropic, a business started by former OpenAI researchers with experience in language models such as GPT-3, created the AI helper Claude (Wu et al., 2023). With a high Google investment, Anthropic seeks to develop AI that is both beneficial and safe. AnthropicLM v4-s3, their flagship model, is an autoregressive model with 52 billion parameters that was trained on enormous text datasets. Anthropic uses a novel “Constitutional AI” technique in contrast to conventional fine-tuning techniques that depend on human input (Bai et al., 2022). This novel system employs a model to direct the process of fine-tuning, guaranteeing that the AI abides by a set of principles centered on autonomy (respecting freedom of choice), beneficence (maximizing positive impact), and non-maleficence (avoiding giving harmful advice).

#### 1.2.7 Open-source LLMs

Open Source Large Language Models (LLMs) stand in contrast to proprietary models like GPT and Claude, which are often fine-tuned to align with human preferences, enhancing their usability and safety. This alignment process, however, can be expensive in terms of computational resources and human annotation, and its lack of transparency can hinder progress in AI safety research within the wider community. Open source LLMs offer an alternative by providing researchers and developers with the ability to examine, modify, and build upon the underlying technology. This fosters innovation, allows for greater customization, and promotes a deeper understanding of these models' inner workings. Llama 2 prioritizes helpfulness and safety through specific training. Qwen modifies the Transformer architecture for efficiency and long sequence handling.

The two notable open source LLMs are Llama 2 and Qwen. Llama 2 is a family of pre-trained and fine-tuned LLMs developed by Meta AI, scaling up to 70 billion parameters. Considering to achieve the two benchmarks, helpfulness and safety, Llama 2-Chat models reportedly outperform existing open-source models on benchmarks for both these qualities and, in human evaluations, appear to be comparable to some closed-source models. Meta AI implemented several safety measures, including the use of safety-specific data for annotation and tuning, red-teaming exercises to identify vulnerabilities, and iterative evaluations to refine safety. The accompanying documentation provides a detailed account of their fine-tuning methodology and their strategies for enhancing LLM safety. A noted limitation of Llama 2, particularly its larger versions, is the longer computational time required for operation.

Qwen, short for Tongyi Qianwen, is another open-source LLM that utilizes a modified version of the Transformer architecture, inspired from the LLama model. Qwen's architecture incorporates several specific modifications. It employs a unified embedding approach, which aims to improve performance at the cost of increased memory usage. For incorporating positional information, Qwen utilizes Rotary Positional Embedding (RoPE). Additionally, biases are added to the Query, Key, and Value layers of the attention mechanism to enhance the model's ability to handle longer sequences. Qwen also replaces the traditional layer normalization technique with RMSNorm, which is reported to offer similar performance with greater efficiency. For the activation function, Qwen has chosen SwiGLU, a combination of Swish and Gated Linear Unit.

Both Llama 2 and Qwen represent significant advancements in the realm of open-source LLMs. They provide transparency and flexibility, enabling the AI community to understand and build upon these technologies. Llama 2 places a strong emphasis on safety through dedicated training and evaluation methodologies, while Qwen introduces architectural modifications aimed at improving performance and efficiency, particularly in handling longer sequences. Both models contribute to the growing landscape of accessible and powerful language models.

## 2 Taxonomy of AI tasks

The usage of LLMs in the context of EEG-based disease diagnostics can be classified into generative and discriminative tasks. Generative tasks help us create all new content, such as textual output showing the reasoning behind a specific decision. On the other hand, discriminative tasks are useful for the categorization of given input data into classes, such as in the case of disease classification. Utilization of both of the models is important for achieving effective disease diagnostics. Use of various key LLM models in the context of neuro signal analysis is listed in Table 1.

**Table 1:** Description of key LLM models in the context of neuro signal analysis.

**Research**	**Year**	**LLM**	**Task**
Mishra et al. (2024)	2024	Llama v3, MISTRALv0.3, QWEN2.5	Generative
Tung et al. (2024)	2024	Gemini 1.5 flash, Claude 3 sonnet, GPT-4	Generative
Chen et al. (2024)	2024	Qwen2-0.5B	Generative
Wang et al. (2024a)	2024	BART	Generative
Kim et al. (2024)	2024	da Vinci GPT-3 base	Discriminative
Parani et al. (2024)	2024	pre trained Longformer	Discriminative
Jiang et al. (2024)	2024	Large Brain Model (LaBraM)	Discriminative
Zhang et al. (2023)	2023	GPT-3.5, GPT-4	Discriminative
Gijsen and Ritter (2024)	2024	EEG Language Model (ELM)	Discriminative
Lee and Chung (2024)	2024	GPT-3.5 turbo model	Discriminative
Sano et al. (2024)	2024	GPT-4, GPT-4 Vision, GPT-3.5	Discriminative
Zhang et al. (2024c)	2024	BERT	Discriminative
Wang et al. (2024b)	2024	Llama 2	Generative
Han et al. (2024)	2024	BERT	Generative
Ma et al. (2024)	2024	miniGPT-4, CLIP	Generative
Yang et al. (2024)	2024	Qwen2 1.5B	Generative
Zhang et al. (2024a)	2024	Llama 2	Generative

**Figure 1:**
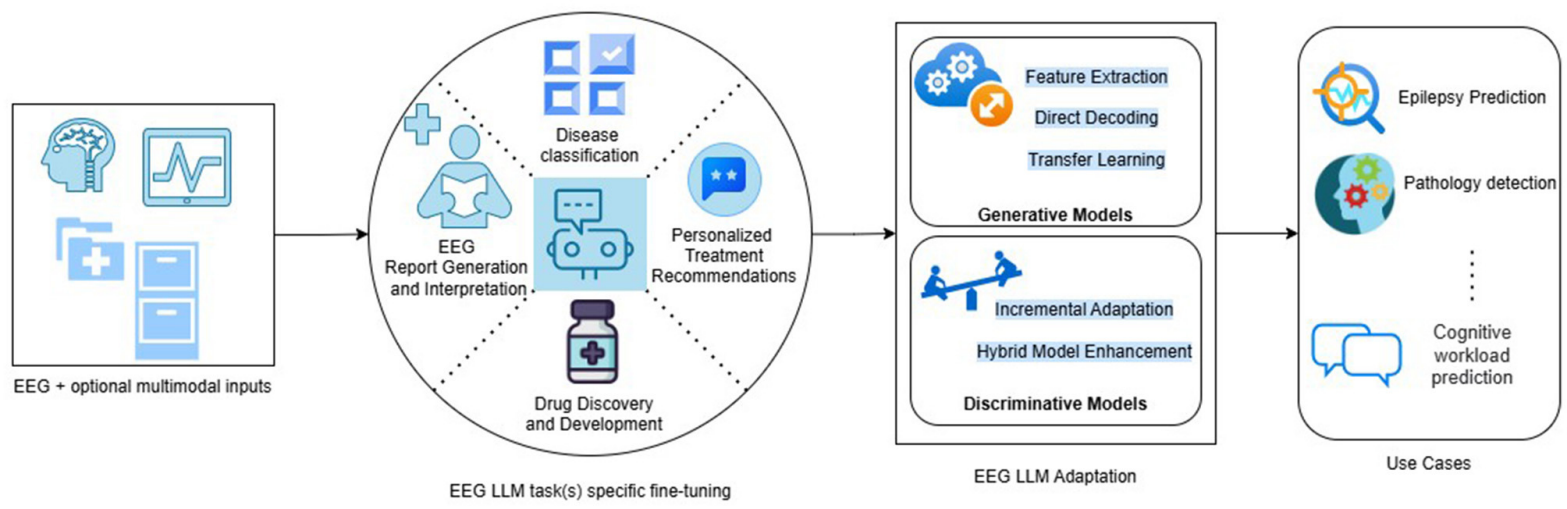
Overview of LLM solutions for EEG diagnosis use cases.

The block diagram shown in Figure 1 details the modules involved in a typical machine-learning pipeline using LLM for EEG-based disease diagnostics. It has got 4 stages as given below:

- Input Stage
  This stage reads the input data in the form of EEG which can optionally be multimodal inputs bringing additional information helpful for categorization or generation of data at the output.
- EEG LLM fine-tuning stage
  Fine-tuning is the key step of the pipeline and tunes the LLM for a specific context which can be a single task or multiple tasks in the same context. This stage uses various adaptation methods for tuning a given LLM for a specific task such as report generation or disease classification. Internally it makes use of techniques such as transfer learning and incremental adaptation for generative tasks, and incremental adaptation and hybrid model enhancement for discriminative tasks. It can be a mixture of both approaches and the associated techniques based on the use case that is being addressed.
- Output stage
  The output can be generated text or labels indicating the class of health condition of the patient. It can be a combination of both for hybrid models. In case of multi tasking, the labels generated by the model can belong to different set of classes based on the task in focus.

### 2.1 LLMs for generative tasks

#### 2.1.1 Thought2Text

The goal of this approach is to evaluate efficiency of public LLMs such as LLAMA v3, MISTRALv0.3, and QWEN2.5 in translating visual thoughts from EEG signals into textual form (Mishra et al., 2024). This is achieved by a 3 step approach, involving capturing of EEG signals, encoding of these signals as token embeddings and fine-tuning of language models with these features.

For the first step, to generate embeddings, this solution makes use off a EEG encoder derived from a deep convolutional neural network model—ChannelNet—that converts EEG signals to multidimensional embeddings (Heeg). Pooled image embeddings (Hclip) are generated by a pre-trained CLIP model capable of abstracting image representations. The encoder functions by minimizing two set of losses—one which is a categorical cross-entropy loss between predicted and actual labels using EEG embeddings and secondly, the mean squared error (MSE) between EEG embeddings (Heeg) and pooled image embeddings (Hclip). In the following stage, these representations are further translated into multimodal embeddings (Hmm) by passing through a projector implementing a transformation. The LLMs learns representation in multimodal feature form generated from an image sketch filtering original image using Gaussian blur and Canny filters. In the next step, it learns the representation generated by the projector using a multichannel EEG signal that represents the response of the brain to the image shown to the subject.

On using this trained model for inference, the EEG encoder generates EEG embeddings and makes use of no images. This representation is further passed through projectors to get multimodal features for performing predictions. The embeddings from EEG signal segments are further concatenated and given to fine-tuned LLM which generates meaningful text descriptions.

#### 2.1.2 Multi-stage LLM report generation

The objective of the proposed system here is to generate and verify EEG reports with the help of a multi-stage LLM solution (Tung et al., 2024). Generation phase, which is first among the two phase approach, takes EEG features as inputs and format them to generate a structured prompt. This prompt is also given to the Google Gemini 1.5 pro API for the processing and generation of reports. In the second phase, the system uses 3 promising LLMs—Gemini 1.5 flash, Claude 3 sonnet, and GPT-4—and performs verification. This solution makes use of advanced LLM capabilities of Gemini pro to build its core language model. The model is chosen for its advanced capabilities that include long context memory, reasoning abilities, and optimized computational performance. The input of this pipeline employs a hybrid AI algorithm that generates a JSON object based on structured EEG features. This ensures capturing of important metrics such as background frequency, amplitude, symmetry, and detected anomalies. These objects are further used by the LLM along with an efficient prompt engineering method. The prompt engineering method used in this system has four parts, namely : (1) role as neurologist, (2) structure EEG features and interpretations, (3) task specifications for generation of report, and (4) outline of report. Each of the LLM model is set to assess independently and finally decision is based on majority voting. Validation of the method by generation of reports on a few hundred report showed effectiveness of the system in guiding neurologist to make infallible decisions.

#### 2.1.3 EEG emotion copilot

EEG emotion copilot uses a lighter LLM in a local server to perform multiple tasks using EEG signals (Chen et al., 2024). The features of this system include emotion recognition, the generation of custom diagnostics, treatment recommendations, and the automatic creation of medical records for patients. It also provides an ergonomic user interface and employs strong privacy safety measures through novel data processing protocols.

The study methodology involves pre-processing EEG signals and transforming them via wavelet to shorten the signal length. The final prompt is constructed using the initial context-defining prompt, demographic data, emotional label, and treatment as training data. Qwen2-0.5B pre-trained model is used for pruning and achieving 50% reduction over the model parameters. A warm-up during the fine-tuning phase using Lora gradually increases the learning rate of the model. Finally, the RAG (Retrieval-augmented generation) technique is used to deploy the model to enhance retrieval performance and improve the interactivity through the dialogue method in the user interface.

This approach addressed the issue of data redundancy inherent in EEG signal processing. The long EEG data sequence handling was managed through efficient data compression techniques, thus improving computational efficiency and computing the real-time emotion. This study highlighted the importance of patient privacy by ensuring that the proposed model is run locally. Model pruning strategies were explored to create a lightweight version of the language model, making it feasible to deploy in environments with limited computational resources while maintaining high performance. While signal compression improves efficiency, complex scenarios still require additional channel signals for accurate analysis. The study proposed that LLMs could potentially generate dense channel signals from limited channel data, which would significantly enhance computational efficiency. This approach could revolutionize emotion analysis and streamline the overall process. This research demonstrates the potential of EEG Emotion Copilot to transform emotional recognition and treatment in clinical settings.

#### 2.1.4 Contrastive EEG-text masked autoencoder

This research work reports a significant advancement in EEG-based language decoding through CET-MAE (Contrastive EEG-Text Masked Autoencoder) and E2T-PTR (EEG-to-Text using Pre-trained Transferable Representations) (Wang et al., 2024a). While E2T-PTR utilizes these pre-trained representations together with BART for better text generation, the CET-MAE model combines masked autoencoding with contrastive learning in an innovative manner for both single-modality and cross-modality processing. This study suggests a novel pre-trained model called Contrastive EEG-Text Masked Autoencoder (CET-MAE) to align EEG and text. CET MAE uses a specialized multi-stream encoder to combine masked signal modeling and contrastive learning. By balancing the semantic-level aligned embeddings of text tokens and text-evoked EEG features with the latent embeddings represented by self-reconstruction, it efficiently learns pre-trained representations of text and EEG. Concerning masked signal modeling, CET-MAE applies a high mask ratio (75%) to both text and EEG data, which poses a significant challenge for the model to manage more missing data during the reconstruction step.

CET-MAE integrates intra- and cross-modal SSL into a single unified system utilizing a multistream architecture: (1) Using masked modeling with a mask ratio of up to 75%, intramodality streams investigate representative embeddings that capture the inherent properties of text or EEG sequences. (2) The intermodality stream constrains the encoder to maximize semantic consistency between text and its related EEG sequences and offers dual-modal representations to improve intramodality reconstruction. E2T-PTR uses BART's capabilities to generate text from these consistent and representative features by transferring pre-trained EEG representations. Multiple experiments using ZuCo, the latest text-evoked EEG dataset, highlight the high standard of this study in both qualitative and quantitative evaluations. Other inner speech BCI data sets can also be used to study the performance of the suggested CET-MAE model, which exhibits significant potential to improve EEG-based language decoding tasks.

#### 2.1.5 LLM analysis of fMRI language data in neurocognitive disorder

A study by Wang et al. (2024b) investigates language-related functional changes in older adults with Neurocognitive Disorders (NCD) using LLM-based fMRI encoding. This work explores the correlation between brain scores derived from fMRI encoding models and cognitive scores in subjects with NCD, in contrast to previous studies that focused on healthy young adults. This study develops an fMRI encoding model using LLaMA2, specifically for older adults with early stage NCD or at risk, in order to quantify the association between brain areas and language functions. Individuals with higher cognitive abilities were revealed to have better brain scores compared to those with lower cognitive abilities, with maximum correlations observed in the middle temporal gyrus (r = 0.368) and the superior frontal gyrus (r = 0.289). This suggests that fMRI encoding models and brain scores have the potential to detect early functional changes in NCD patients, offering a promising avenue for developing interpretable machine learning models for early detection of NCD based on language-related fMRI signals. This study marks the beginning of applying an LLaMA2-based fMRI encoding model to study subjects with NCD.

#### 2.1.6 Mindformer

Mindformer, introduced by Han et al. (2024), is a novel semantic alignment method for multisubject fMRI signals, designed to overcome limitations in current multisubject brain decoding techniques. MindFormer generates fMRI-conditioned feature vectors suitable for conditioning Stable Diffusion for fMRI-to-image generation and LLMs such as Bidirectional Encoder Representations from Transformers (BERT) for fMRI-to-text generation. The model incorporates two key innovations: subject-specific tokens to capture individual differences while leveraging multisubject data for training, and a feature embedding and training scheme based on the Image Prompt Adapter(IP)-Adapter to extract semantically meaningful features from fMRI signals. By effectively embedding multisubject fMRI signals using subject tokens and the IP-Adapter, MindFormer significantly outperforms existing multisubject brain decoding frameworks. This advancement provides a new framework for understanding the decoding of the brain of multiple subjects and identifying common neural patterns, effectively leveraging shared information while maintaining individual-specific accuracy. The current implementation primarily focuses on visual stimuli, and extending it to more complex cognitive and sensory experiences requires advancements in model architecture and training methodologies. However, the computational complexity associated with training on larger datasets presents a limitation.

#### 2.1.7 LLM visual encoding model

LLM Visual Encoding Model (LLM-VEM) introduced in Ma et al. (2024) provided a new multimodal training paradigm, utilizing miniGPT-4 to enhance the encoding of fMRI activity in the visual cortex. The paradigm generates detailed textual descriptions for stimulus images using the LLM, creating a high-quality text description set. These descriptions are then processed through a pre-trained text encoder, namely Contrastive Language Image Pre-training (CLIP), to obtain text embedding features. A contrastive loss function is used to minimize the distance between image embedding features and text embedding features, aligning the stimulus image and text information. This alignment, facilitated by the LLM, improves the visual encoding model learning process, leading to higher precision. Such an effective visual encoding model helps researchers investigate and predict the brain responses to different visual stimuli.

LLM-VEM processes stimulus image features in two stages: Stage 1 utilizes a frozen image feature extractor, Explore the limits of Visual representation at scAle (EVA), for feature extraction, followed by dimensionality reduction via feature projection. To mitigate overfitting, a portion of the voxel mapping network is replaced with a Principal Component Analysis (PCA) module, reducing model parameters. Stage 2 refines the model by unfreezing specific blocks within EVA while freezing others, and incorporates the LLM-aligned loss function to further align stimulus image and text features. By extending unimodal features to multimodal features, this training paradigm improves the encoding model performance. LLM-VEM integrates stimulus images and textual descriptions, aligning them to obtain multimodal feature information and achieve strong performance.

#### 2.1.8 NeuGPT

NeuGPT is a multimodal language generation model designed to unify the analysis of various neural recording types (EEG, MEG, ECoG, SEEG, fMRI, and fNIRS) which have traditionally been studied separately (Yang et al., 2024). The goal is to create a model that can process various neural signals and interact with speech and text, focusing on brain-to-text decoding.

The model is structured in two main stages:

- Stage 1: Neural signal tokenization: this stage focuses on converting neural signals into discrete codes. It consists of four components: an encoder that transforms raw neural signals into embeddings, a quantizer that converts these embeddings into discrete code indices, a decoder that reconstructs the neural signals from the quantized embeddings, and a discriminator that enhances the quality of the reconstructed signals.
- Stage 2: LLM fine-tuning for neural code understanding: this stage involves fine-tuning a large language model (LLM) to understand and generate neural codes, facilitating cross-modal communication between neural signals, speech, and text. QWEN2-1.5B, a relatively small but efficient LLM with a 32K context length, was chosen as the base model for this fine-tuning.

This model demonstrates the feasibility of translating neural signals into coherent speech and text, bridging the gap between brain activity and expressive communication. Highlights the benefits of a unified framework for processing various types of neural signal, overcoming the traditional compartmentalization in neural recording research. The model's flexibility in handling various sensor layouts and coordinates allows for broader application across different experimental settings. The integration of neural signals into language generation models offers insight into human brain language processing and paves the way for advanced brain-computer interfaces.

#### 2.1.9 fNIRS and LLM for VR rehab evaluation in mild cognitive impairment

The study addresses the challenge of effectively evaluating Virtual Reality (VR) tasks designed for Mild Cognitive Impairment (MCI) rehabilitation (Zhang et al., 2024b). Traditional evaluation methods, such as post-training metrics and subjective questionnaires, do not capture the comprehensiveness and intensity of cognitive stimulation provided by VR tasks. To overcome these limitations, Zhang et al. (2024b) proposed a novel approach that integrates functional near-infrared spectroscopy data with an LLM to evaluate and optimize VR rehabilitation tasks.

The study introduces a systematic paradigm, based on the Diagnostic and Statistical Manual of Mental Disorders (DSM-5), to assess the scope of cognitive domains stimulated by VR tasks. This paradigm enables a unified assessment of various cognitive domains, including attention, memory, executive functions, language, visuospatial skills, and psychomotor abilities. This study uses fNIRS technology to objectively measure cognitive stimulation with high time resolution. They extract graph parameters from fNIRS data to quantify brain region connectivity and efficiency during VR tasks, providing robust neural indicators of cognitive engagement.

**LLM-enabled analysis**: A key innovation of the study is the development of a three-stage prompt strategy to facilitate LLM-based analysis. The LLM is used to translate complex metrics derived from fNIRS and the scope of stimulated cognitive domains into easy-to-understand evaluation reports and actionable recommendations for VR task optimization. This approach aims to bridge the gap between complex neural observations and practical insights for VR task designers.

This approach exhibits the potential of integrating fNIRS data and LLMs to provide a comprehensive and objective evaluation of VR rehabilitation tasks. The proposed framework improves the design and effectiveness of VR interventions for MCI, by automating the analysis and interpretation of complex neural data.

#### 2.1.10 MindSpeech

A novel AI model, named MindSpeech, is designed to decode imagined continuous speech using high-density functional near-infrared spectroscopy (fNIRS). The study aims to develop a non-invasive brain-AI interface that can translate imagined thoughts into text, enhancing human-AI communication.

Zhang et al. (2024a) used high-density fNIRS to record brain signals from participants engaged in an imagined speech task. They developed a “word cloud” paradigm to elicit a variety of imagined sentences across a broad semantic space. In this paradigm, participants were presented with a central topic word and surrounding keywords and instructed to imagine sentences using these words. After the imagined speech period, the participants typed the sentences, providing ground-truth data for decoder training. In addition, a continuous-wave high-density fNIRS system was used to collect neurovascular data. The fNIRS data was preprocessed through several steps, including conversion to optical density, detrending, motion artifact removal, and bandpass filtering.

The core of the MindSpeech model involves using a prompt tuning approach with the Llama2 model. This approach allows the LLM to generate text guided by the fNIRS brain signals. The process includes segmenting the imagined sentences into context input and continuation, converting both context input and fNIRS signals into embeddings, and concatenating these embeddings as input to the LLM.

A brain encoding model, using a sequence-to-sequence (Seq2Seq) neural network with transformers, maps the fNIRS data to LLM embeddings. The model is trained to predict the continuation text from the brain signal-generated embeddings and the context input embeddings. The model's performance was evaluated using natural language processing metrics to compare the generated sentences with the ground truth. The study also explored the combination of data from multiple participants to improve the decoder performance.

#### 2.1.11 Language postdiction vs. prediction in MEG

A research study by Azizpour et al. (2024) investigated whether MEG data can reveal predictive information during natural listening, similar to findings in fMRI. The researchers examined whether pre-onset neural encoding of upcoming words could be detected in MEG signals, aligning with results from other neuro signals. They also tested whether incorporating future word embeddings, as done in fMRI studies, would enhance the alignment between MEG data and linguistic predictions. To address these questions, the study built encoding models using GPT-2 embeddings to map to MEG data recorded while participants listened to approximately 10 h of narrated stories. The results showed that the GPT-2 embeddings explain the variability in post-onset MEG signals. Critically, consistent with electrocorticography findings, pre-onset representations of upcoming words were detected up to 1 second before word onset in language-related regions. However, unlike fMRI findings, including future word embeddings did not improve MEG encoding.

The study concludes that while MEG can capture pre-onset representations similar to electrocorticography, the lack of enhancement with future word embeddings suggests that these signals might not reflect predictive processing and could be due to correlations between nearby embeddings and word co-occurrences. The findings also revealed robust evidence for postdiction. In general, the study demonstrates the value of MEG combined with LLMs for studying naturalistic language processing and emphasizes the need for more research to define evidence for prediction in this context.

### 2.2 LLMs for discriminative tasks

#### 2.2.1 EEG-GPT

EEG GPT is an attempt to use a comparatively small training data set to fine-tune an LLM and achieve performance comparable to that of other classical approaches in a deep learning context, for classification of the given EEG signal segment as normal or disease. It shows that with zero-shot learning, the base LLM yields improved performance in such classification tasks (Kim et al., 2024). The pipeline used for this approach generates quantitative EEG features that are fed to a fine-tuned LLM that uses a specific private knowledge base. The dataset used here is the Temple University Hospital Abnormal Corpus, which is made up of 1140 hours of EEG data acquired from 2,993 subjects. It is balanced between normal and abnormal recordings to some extent and is further pre-split into train and evaluation sets for uniformity of evaluation over experiments.

Each of the given EEG files is segmented into non-overlapping 20-second epochs and quantitative features such as standard deviation, kurtosis power ratios, etc. are calculated for each epoch. Open AI's Completions APIs are further used to fine-tune and evaluate on da Vinci GPT-3 base LLM. The original quantitative features are converted to verbal representation with the use of prompts, making it generate normal/disease labels at the output. This solution aims to use 50 times less data, yet provides performance comparable to that of deep learning approaches such as ChronoNet, StanfordCNN, and HybridCNN. This study also highlights the reasoning ability and ability of EEG GPT to make use of specialist EEG tools on several temporal scales in a gradual and transparent way. Such a tree-of-thought reasoning approach helps generate the reasoning behind predictions in a human-readable form by making use of tools such as qEEG. By going through multiple segments until the system is confident in predicting the start and duration of a seizure or as normal, this solution helps to allow early stoppage of seizures.

#### 2.2.2 Pretrained longformer LLM

This method utilizes a large language model for epilepsy classification by re-factoring the data for a pre-trained LLM model (Parani et al., 2024). Such an approach requires minimal retraining and still results in better performance compared to deep learning models trained from scratch.

The data preparation stage of the mode converts EEG signals that are in the form of real numbers into string tokens. Due to memory limitations, the tokenization is performed on each of the 20 EEG channels individually. The generated tokens are divided into segments corresponding to 1 second each to improve efficiency. Further, a locally deployed open-source LLM—Longformer—is utilized for learning from generated features, i.e., tokens. By using sliding window attention to process tokens within a specific window and symmetric global attention that captures relationships between pairs of tokens, LLM is trained for the given disease classification context. The training is focused on the classifier layer of Longformer with a chosen set of hyperparameters for 4 batches. The final classification result of a segment corresponding to detection or otherwise is performed by majority voting, where detection over 10 channels indicates a positive label.

The study further compares the chosen LLM solution against ViT methods having multiple stages of transformer blocks followed by a classifier stage. ViT is efficient in extracting spatial features and short-term local temporal features efficiently. However, its incapability to capture long-term temporal dependencies and correlations makes it inferior to the aforementioned LLM method of disease classification.

#### 2.2.3 NeuroLM—multitask foundation model

Even though there are many advances in large-scale pre-training with EEG, proving significant potential for advancing brain-computer interfaces and healthcare applications, current pre-trained models typically require complete fine-tuning for each downstream task (Jiang et al., 2024). This limits their flexibility and leads to inefficient resource usage. This study develops NeuroLM, a multi-task foundation model that treats EEG signals as a foreign language, leveraging the capabilities of Large Language Models (LLMs) to enable multi-task learning and inference. NeuroLM addresses three major challenges in combining EEG processing with LLMs: the alignment of EEG and text embeddings, effective representation learning within the LLM framework, and unified multi-task learning across diverse EEG applications. This system introduces a text-aligned neural tokenizer that converts EEG signals into discrete neural tokens through vector-quantized temporal-frequency prediction. These tokens are then processed by an LLM that learns causal EEG information through multi-channel autoregression and enables the model to understand both EEG and language modalities.

The architecture of this model is remarkable for its scale and comprehensive training approach. It features 1.7B parameters which have been pre-trained on approximately 25,000 h of EEG data. The data goes into a text-aligned neural tokenizer which is trained through adversarial training. In the next step, a VQ encoder helps extract compressed embedding representations for LLM processing. Finally, multitasking instruction tuning helps to implement a vast set of downstream applications.

The dataset for the study included six different EEG datasets to evaluate NeuroLM, TUAB (Harati et al., 2015) (abnormal detection), TUEV (Zheng and Lu, 2015) (event type classification), SEED (Zheng and Lu, 2015) (emotion recognition), HMC (Alvarez-Estevez and Rijsman, 2021) (sleep stage classification), Workload (Zyma et al., 2019) (cognitive workload classification) and TUSL (von Weltin et al., 2017) (slowing event classification). The model performance is demonstrated across six different tasks, including abnormal detection, event type classification, emotion recognition, sleep stage classification, cognitive workload prediction, and slowing type classification. The use of instruction tuning for multi-task learning in EEG signal processing has shown remarkable success in this model, thus eliminating the need for individual fine-tuning while maintaining high performance across various applications.

#### 2.2.4 Word-level neural state classification

This study makes use of LLMs that are provided by eye-tracking data and EEG measurements, for the investigation of neural responses (Zhang et al., 2023). It utilizes the Zurich Cognitive Language Processing Corpus (ZuCo), focuses on semantic inference processing and analyzes brain states during word fixation periods.

The classification pipeline consists of (i) Initial word classification where two language models evaluate sentences and words categorized into: high-relevance words (HRW) and low-relevance words (LRW), (ii) Data processing where joint selection process identifies shared HRW set, eye-gaze data is used to extract corresponding EEG signals and four feature-extraction techniques are applied to reduce signal complexity, and (iii) Classification System where three distinct classifiers implemented and follow standard brain-computer interface methodology to perform binary HRW/LRW classification. It achieved over 60% validation accuracy across 12 subjects and successfully distinguished between high and low-relevance word processing. This is the first study to classify brain states at the word level using LLM knowledge and contributes to the understanding of human cognitive processing.

#### 2.2.5 Zero-shot pathology detection

The study integrates clinical EEG data with language modeling and develops a novel approach for medical diagnostics and pathology detection, based on an extensive dataset of 15,000 EEGs paired with corresponding clinical reports (Gijsen and Ritter, 2024). It employs contrastive learning techniques and is one of the pioneer works in applying multimodal pre-training using natural language and functional brain data in a medical context. It seems that exposure to a range of textual material combined with contrastive learning produces the most accurate representations. In particular, retrieval performance was significantly enhanced by integrating data on the patient's medication and clinical history with EEG interpretation. Zero-shot pathology detection also proved to be possible with such multimodal models. It showed significant performance over EEG-only SSL was noted using linear probing, with the greatest improvements in situations with a limited number of annotated samples.

#### 2.2.6 LLM for neural decoding

This study aims to employ LLM to develop a novel neural decoder for interpreting intracranial EEG (iEEG). It tried to overcome the limitations of traditional decoders, which often specialize in specific tasks and struggle to interpret complex, real-world brain activity (Lee and Chung, 2024).

This novel approach can provide more comprehensive, and faster interpretations of iEEG signals more efficiently. The GPT-3.5 turbo model was fine-tuned with preprocessed iEEG signals categorized by frequency bands [high-gamma (30–200 Hz), beta (12–30 Hz), and theta (4–8 Hz)] and by the regions of the brain. These signals were presented as prompts to the model. A Python-based system was developed to integrate neural signal processing with the LLM decoder. The authors observed frequent responses corresponding to visual and auditory stimuli. This variability in responses to identical prompts highlights a limitation, which could be addressed through more specific fine-tuning of the LLM.

#### 2.2.7 LLM on human attention

This research applies LLMs in the context of human attention and sleep and tries to estimate the stages and quality of sleep and attention states (Sano et al., 2024). The model can generate suggestions for improving sleep and adaptive guided imagery scripts based on electroencephalogram (EEG) and data related to physical activity. This study's results show that LLMs can estimate sleep quality based on human textual behavioral features, even though it requires further training data and domain-specific knowledge. The study utilized (a) zero-shot learning: LLMs (GPT-4, GPT-4 Vision) were used without specific training, relying on their pre-trained knowledge to interpret the input data. (b) In-context learning: LLMs (GPT-4) were provided with input data and label examples within the prompts to enable them to learn from the context. (c) Fine-tuned LLMs: GPT 3.5 Turbo was fine-tuned on specific datasets for improved performance, and (d) traditional Machine Learning: XGBoost, a gradient boosting algorithm, was used as a benchmark for comparison. The study focused on using interpretable features (e.g., power spectrum density) to understand the extent to which LLM contributes to the detection and improvement of altered states of sleep.

LLMs, even with fine-tuning, showed lower accuracy in directly detecting attention states, sleep stages, and sleep quality from EEG and activity data compared to traditional machine learning models like XGBoost. This study is done with limited datasets and limited LLMs. Refining prompts and using large and diverse datasets can enhance the model's performance. More extensive training of LLM can be done with diverse physiological and behavioral data to effectively capture complex human patterns.

#### 2.2.8 LLM on human reading comprehension

This study developed a Brain-Computer Interface (BCI) system that can predict the relevance of words during reading comprehension tasks by integrating EEG and eye-tracking data with a novel reading embedding representation. LLMs are used to guide the learning process and understand the underlying semantic relationships within the text (Zhang et al., 2024c). This study uses the pre-trained BERT model to generate word embedding that helps to learn the semantic context of every token within a given sentence. In addition, it also utilizes important eye-gaze features such as fixation duration and pupil size, as well as conditional entropy of the EEG signal at the input. In the next step, these bio-signal features are normalized and projected into a common space. The final set of processed features is passed on to an attention-based transformer encoder combining word embeddings and biosignal features resulting in effective multimodal representations. This approach provides a reliable LLM-guided labeling process.

This improvement highlights the superior performance of the transformer architecture in handling complex, multi-modal data. This representation, which combined eye-tracking and EEG biomarkers using an attention-based transformer encoder, had the highest single-subject accuracy of 71.2% and a mean 5-fold cross-validation accuracy of 68.7% across nine people using a balanced sample. This is a pioneer study in eye tracking, EEG, and LLMs to predict human reading comprehension at the word level. Without any prior information about the reading tasks, the Bidirectional Encoder Representations from Transformers (BERT) model is fine-tuned for word embedding. The model easily achieves an accuracy of 92.7% despite the lack of task-specific information.

## 3 Discussion

The studies considered here show the ability of LLMs to generate several meaningful features, especially proving to be promising for use cases where the available data set size is limited. This is achieved through the use of zero-shot and few-shot learning. Recent research has shown the effectiveness of LLMs in performing few shot learnings in domains ranging from seizure forecasting to EEG textual report generations. Many of these works have reported that these transformer architectures are efficient in making use of in-context learning for zero-shot tasks, merely by utilization of information given over a textual prompt resulting in better performance for both generative and discriminative tasks.

One of the key advantages of LLMs is their ability to generate intermediate reasoning steps for the analysis of complex problems. The use of strategies such as Chain of Thought for multi-step calculations with LLM was proven to be effective due to strategic lookahead and backtracking. Moreover, LLMs are proven to be capable of using external expert tools in the analysis of EEG and then synergizing those outputs to generate more meaningful results, similar to a subject matter expert of the domain.

### 3.1 Ethical considerations

A serious aspect of using LLMs with neurological signal processing and analysis is its ethical considerations. The data, that is fed to the LLMs are personal physiological and behavioral data which can raise privacy concerns. The users might get worried about security and confidentiality, as the data is sent to cloud servers with the use of popular LLMs such as GPT 4, Gemini, and Claude. Transparency and effective data anonymization are essential in this regard for avoiding issues due to leakage of data from cloud platforms. Additionally, concerns are raised around the generated contents from LLMs, which potentially be harmful and inaccurate or may intend to manipulate the user. To avoid this, implementing comprehensive guidelines covering ethical and safety aspects is necessary.

A possible solution to reduce concerns around privacy and security is to run the models locally on high-end servers. However, this requires model pruning resulting in lightweight LLMs well-suited for local execution with limited resources. Such a solution often results in compromises around model's effectiveness in terms of prediction accuracy and computational time.

### 3.2 Limitations and future work

The need for LLM in the context of EEG analysis arises from the gaps that were identified from existing literature. One major challenge is the limited availability of EEG data. Unlike image or text data collection, acquiring EEG data is complex. Expert annotation is particularly time-consuming and results in small datasets of labeled EEGs for specific BCI tasks. Existing EEG datasets are not substantial enough to support robust LLM training required for significant model efficiency gains. Thus the questions to address are: how can we effectively utilize large-scale unlabeled EEG data, and what volume of data would be necessary for training LLMs?

Varying EEG collection configurations pose another challenge to the use of LLMs for EEG analysis. Although the international 10–20 system provides standardization guidelines for EEG testing, clinicians often use different numbers of electrodes based on their specific application requirements. This variability creates a significant research challenge in adapting various EEG data formats to align with the input specifications of the neural transformer.

An additional hurdle involves developing effective EEG representation learning approaches. The primary difficulty is the low signal-to-noise ratio (SNR) and various types of interference. Successfully balancing temporal and spatial characteristics is essential for effective learning of EEG representation. Despite the existence of various deep learning approaches for raw EEG data processing, including CNN, RNN, and GNN architectures, many researchers continue to rely on manually designed EEG features due to these inherent challenges.
